# Role of Estrogen Receptors α and β in a Murine Model of Asthma: Exacerbated Airway Hyperresponsiveness and Remodeling in ERβ Knockout Mice

**DOI:** 10.3389/fphar.2019.01499

**Published:** 2020-02-04

**Authors:** Rama Satyanarayana Raju Kalidhindi, Nilesh Sudhakar Ambhore, Sangeeta Bhallamudi, Jagadish Loganathan, Venkatachalem Sathish

**Affiliations:** Department of Pharmaceutical Sciences, School of Pharmacy, College of Health Professions, North Dakota State University, Fargo, ND, United States

**Keywords:** estrogen receptor alpha, sex difference, mixed allergen, flexiVent, fibronectin, collagen, vimentin, α-smooth muscle actin

## Abstract

Epidemiological data suggests increased prevalence of asthma in females than males, suggesting a plausible role for sex-steroids, especially estrogen in the lungs. Estrogen primarily acts through estrogen-receptors (ERα and ERβ), which play a differential role in asthma. Our previous studies demonstrated increased expression of ERβ in asthmatic human airway smooth muscle (ASM) cells and its activation diminished ASM proliferation *in vitro* and airway hyperresponsiveness (AHR) *in vivo* in a mouse (wild-type, WT) model of asthma. In this study, we evaluated the receptor specific effect of circulating endogenous estrogen in regulating AHR and remodeling using ERα and ERβ knockout (KO) mice. C57BL/6J WT, ERα KO, and ERβ KO mice were challenged intranasally with a mixed-allergen (MA) or PBS. Lung function was measured using flexiVent followed by collection of broncho-alveolar lavage fluid for differential leukocyte count (DLC), histology using hematoxylin and eosin (H&E) and Sirius red-fast green (SRFG) and detecting αsmooth muscle actin (α-SMA), fibronectin and vimentin expression using immunofluorescence (IF). Resistance (Rrs), elastance (Ers), tissue-damping (G) and tissue-elasticity (H) were significantly increased, whereas compliance (Crs) was significantly decreased in WT, ERα KO, and ERβ KO mice (males and females) challenged with MA compared to PBS. Interestingly, ERβ KO mice showed declined lung function compared to ERα KO and WT mice at baseline. MA induced AHR, remodeling and immune-cell infiltration was more prominent in females compared to males across all populations, while ERβ KO females showed maximum AHR and DLC, except for neutrophil count. Histology using H&E suggests increased smooth muscle mass in airways with recruitment of inflammatory cells, while SRFG staining showed increased collagen deposition in MA challenged ERβ KO mice compared to ERα KO and WT mice (males and females), with pronounced effects in ERβ KO females. Furthermore, IF studies showed increased expression of α-SMA, fibronectin and vimentin in MA challenged populations compared to PBS, with prominent changes in ERβ KO females. This novel study indicates ERβ plays a pivotal role in airway remodeling and AHR and understanding the mechanisms involved might help to surface it out as a potential target to treat asthma.

## Introduction

Asthma is a chronic respiratory disorder causing significant morbidity and mortality worldwide. It is an intricate disorder involving diverse pathophysiologies affecting respiratory structure and thereby function (Townsend et al., 2012a; Prakash, 2013; Raju et al., 2014; Prakash, 2016; Raju et al., 2016). Asthma is characterized by inflammation and remodeling in the airways contributing to airway hyperresponsiveness (AHR) leading to episodic bronchoconstriction’s (Holgate et al., 2009; Prakash, 2013; Prakash and Martin, 2014; Sathish et al., 2015b; Prakash, 2016). Epidemiological data suggests a role of sex in a variety of lung diseases, especially asthma (Riffo-Vasquez et al., 2007; Antunes et al., 2010; Bonds and Midoro-Horiuti, 2013; Lingappan et al., 2016; Wang et al., 2016; DeBoer et al., 2018; Fuentes and Silveyra, 2018; Fuentes and Silveyra, 2019a; Naeem and Silveyra, 2019).

In addition to genetic and environmental factors, sex/gender difference plays a pivotal role in the pathophysiology of asthma (Clough, 1993; Weiss and Gold, 1995; de Marco et al., 2000; Caracta, 2003; Carey et al., 2007b; Card and Zeldin, 2009; Woods et al., 2010; Townsend et al., 2012a; Cabello et al., 2015; Mishra et al., 2016; Fuentes et al., 2018; Fuentes and Silveyra, 2019b; Kalidhindi et al., 2019b). Incidence of asthma is more common in pre-pubescent boys and adult women and the severity of asthma is increased during pregnancy (de Marco et al., 2000; Caracta, 2003; Carey et al., 2007b; Bonds and Midoro-Horiuti, 2013). In this context, multiple studies have explored and suggested a role for sex hormones in airway biology, especially estrogen (Keselman and Heller, 2015; Sathish et al., 2015a; Ambhore et al., 2018; Fuentes and Silveyra, 2018; Ambhore et al., 2019a; Ambhore et al., 2019b; Bhallamudi et al., 2019; Fuentes et al., 2019; Fuentes and Silveyra, 2019a). However, there is still a debate on the contradicting role of estrogen as few studies suggest its role in reducing inflammation (Myers and Sherman, 1994; Lieberman et al., 1995; Haggerty et al., 2003), while others suggest estrogen to induce AHR and inflammation (Riffo-Vasquez et al., 2007; Sakazaki et al., 2008). This warrants a more meticulous study involving receptor specific signaling of estrogen to understand its role in the pathophysiology of asthma.

Estrogen primarily acts through estrogen receptors (ER) ERα and ERβ, which are widely considered to be nuclear receptors (Altucci and Gronemeyer, 2001). Multiple studies suggest both ERα and ERβ signal through different cell signaling pathways eliciting different effects on cellular functions (Kuiper et al., 1996; Clarke et al., 2003; Heldring et al., 2007; Edvardsson et al., 2011; Williams and Lin, 2013; Aravamudan et al., 2017; Ambhore et al., 2018; Ambhore et al., 2019b; Bhallamudi et al., 2019; Fuentes and Silveyra, 2019a). ER’s are expressed on a wide array of cells and our group has recently showed differential ER expression in human ASM cells, which is upregulated during inflammation/asthma (especially ERβ) (Aravamudan et al., 2017). Furthermore, we have also shown that ERβ activation inhibits PDGF induced proliferation in primary human ASM cells *via* AKT/ERK/p38 pathways (Ambhore et al., 2018). Many studies have shown the impact of ER signaling on asthma *in vitro* (Townsend et al., 2010; Townsend et al., 2012b; Martin et al., 2015; Sathish et al., 2015a; Aravamudan et al., 2017; Ambhore et al., 2018; Ambhore et al., 2019b; Bhallamudi et al., 2019); however, very few studies have explored the role of estrogen in asthma *in vivo* (Carey et al., 2007a; Carey et al., 2007c; Riffo-Vasquez et al., 2007; Dimitropoulou et al., 2009; Jia et al., 2011). Few studies reported down-regulated AHR upon administration of estrogen in females and OVX mice; however, the receptor-specific role of estrogen has not been explored (Riffo-Vasquez et al., 2007; Matsubara et al., 2008; Dimitropoulou et al., 2009). Our recent study in wild type C57BL6/J mice shows that ERβ activation using pharmacological agonists alleviates AHR and airway remodeling in a mixed allergen-induced mouse model of asthma (Ambhore et al., 2019a).

Although, pharmacological agonists specifically activate the respective receptors, there happen to be various artifacts influencing the outcomes. In the advent of recent advancements in genome engineering and the arrival of receptor-specific knockout mice, we wanted to confirm our hypothesis using ERα and ERβ specific knockout mice. In the context of estrogen receptor-specific knockout and its effect on asthma, Carey et al. (2007a) have performed a study in ER specific knockout mice, but have mainly focused on ERα KO mice and have reported limited data on ERβ KO mice. An interesting fact to note here is that ERβ expression is increased multifold during asthma when compared at baseline (Aravamudan et al., 2017), justifying the need for *in vivo* study in the context of asthma. Considering these facts, we performed a comprehensive study to identify the role of ER specific signaling of endogenous estrogen during asthma in a mixed allergen (MA) induced murine model of asthma in ER specific knockout mice (ERα and ERβ). In this study, we found that ERβ knock out mice show exacerbated AHR and remodeling, while ERα knock out mice show reduced AHR and remodeling upon MA challenge when compared to wild type mice. Interestingly, in comparison between male and female mice, females from all study population showed a higher degree of AHR and airway remodeling compared to males.

## Materials and Methods

### Animals

Animal study protocol in this study was approved by the Institutional Animal Care and Use Committee at North Dakota State University and conducted in accordance with guidelines derived from the National Institutes of Health’s Guide for the Care and Use of Laboratory Animals. ERα (Stock No: 004744, B6.129P2-Esr1^tm1Ksk^/J) and ERβ (Stock No: 004745, B6.129P2-Esr2^tm1Unc^/J) knock out heterozygous breeding pairs of C57BL/6J background were procured from Jackson Labs (Bar Harbor, ME). All the mice used in this study were homozygous obtained from in-house breeding using ERα or ERβ knock out heterozygous breeding pairs. Obtained litters were separated based on genotyping and the resultant wild type mice and knockout mice were used for the study. Mice were always housed under constant temperature and 12 h light and dark cycles provided with food and water *ad libitum*. Mice from either gender were used in this study with a minimum of 5–6 mice in each group.

### Genotyping

**Figure 1:**
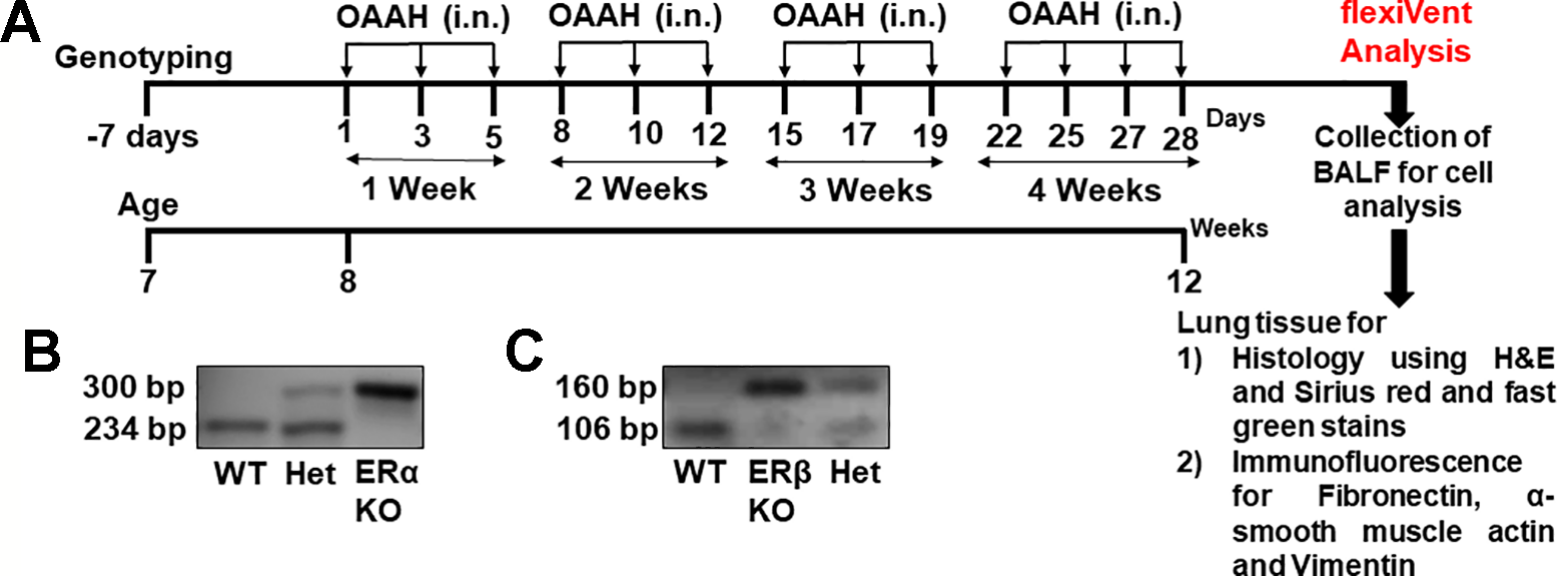
**(A)** Experimental design of the study. Genotyping was performed at the age of 7 weeks and mice were grouped according to their genotype. Mixed allergen (OAAH: 10 μg each of Ovalbumin, Alternaria Alternata, Aspergillus Fumigatus and Dermatophagoides farinae (house dust mite; i.n., intranasal) was administered on alternate days for 28 days while control mice received phosphate buffer solution as vehicle. **(B)** Representative image of ERα KO genotyping. **(C)** Representative image of ERβ KO genotyping.

The pups obtained from the breeding process were subjected to genotyping after 7 weeks using a tail biopsy method following instructions provided by Jackson laboratories (Bar Harbour, ME). The genomic DNA from mouse-tails were isolated by a hotshot method. Briefly, the collected tail snips were homogenized in alkaline buffer (75 µl of 25mM NaOH and 0.2mM EDTA pH 8 solution) followed by heating at 95 °C for 30 min and immediately cooling at 4°C for 15 min. Later Neutralizing buffer (75µl of 40mM Tris HCL) was added and the resultant DNA was used for PCR using following primer sequences; for ERα (WT 5′GTAGAAGGCGGGAGGGCCGGTGTC-3′, Common 5′-TACGGCCAGTCGGGC ATC-3′, Mutant 5′-GCTACTTCCATTTGTCACGTCC-3′) and ERβ (WT 5′-GTTGTGCCAGCCCT GTTACT-3′, Common 5′- TCACAGGACCAGACACCGTA-3′, Mutant 5′- GCAGCCTCTGTTC CACATACAC-3′). The obtained cDNA then subjected to agarose gel electrophoresis in a 2% gel and viewed in a LICOR gel imaging station. Mice DNA samples showing 2 bands (300 bp and 234 bp for ERα and 160 bp and 106 bp for ERβ) were designated as heterozygous, samples showing a single band at 160 bp for ERβ and 300 bp for ERα were designated as Knockout and DNA samples showing 234 bp and 106 bp were designated as wild type (**Figures 1B**, **C**).

### Mixed Allergen (MA) Exposure

Mice allotted to mixed allergen (MA) group were administered intranasally with a mixture of equal amounts (10 μg) of ovalbumin (Sigma Aldrich, USA), and extracts from *Alternaria alternata*, *Aspergillus fumigatus,* and *Dermatophagoides farinae* (Greer labs, USA) for 4 weeks in phosphate-buffered saline (PBS), while PBS alone was administered as a vehicle for 28 days on every alternate day (**Figure 1A**) (Iijima et al., 2014; Yarova et al., 2015; Ambhore et al., 2019a; Britt et al., 2019; Loganathan et al., 2019).

### Lung Function Using Flexivent

All mice were subjected to flexiVent (Scireq, Montreal, Canada) analysis on day 28 to determine respiratory resistance (Rrs), compliance (Crs), elastance (Ers), tissue damping (G) and tissue elastance (H) according to previously published techniques (Aravamudan et al., 2012; Yarova et al., 2015; Ambhore et al., 2019a). The flexiVent based lung function analysis in murine models works similar to spirometry used to analyze lung function in humans, except for the fact that it is an invasive method (Devos et al., 2017). Male and female (WT, ERαKO and ERβKO) mice were anesthetized using ketamine and xylazine (100 mg/kg and 10 mg/kg *i.p.* respectively) and immediately ventilated mechanically using flexiVent system. Respiratory resistance (Rrs), elastance (Ers), compliance (Crs), tissue elastance (H) and tissue damping (G) and were measured and recorded at baseline (0 mg/mL Methacholine, MCh) followed by increasing doses of nebulized MCh (6.25, 12.5, 25.0, 50.0 mg/ml, respectively) delivered at 5 min intervals. The body temperature of mice was consistently maintained at 37°C with a heating pad placed underneath the mice and a bulb placed above at a 45° angle. Electrocardiogram (ECG) was monitored throughout the procedure. Mice were euthanized with an overdose of pentobarbital at the end of the experiment followed by a collection of broncho-alveolar lavage fluid (BALF). Following this, lungs were inflated with Carnoy’s solution (100% Ethanol, Chloroform, Glacial acetic acid in a ratio of 6:3:1 with added ferric chloride) and used for histology and immunofluorescence studies.

### Total and Differential Leukocyte Count in BALF

Estimation of total and differential leukocyte count (DLC) in BALF was performed following previously published methods (Thompson et al., 1996; Raju et al., 2014; Ge et al., 2015; Ambhore et al., 2019a; Kalidhindi et al., 2019a). BALF was centrifuged at 2000 rpm for 5 min at 4°C and the supernatant was discarded. The resultant cell pellet was re-suspended in 100 µl of PBS and total leukocyte count was performed using Countess-II FL cell counter (ThermoFisher, USA). Following this, a smear was prepared using cytospin and the air dried smeared slide was stained with Differential Quick Staining Kit (Modified Giemsa, EMS, USA) and washed with distilled water for 8 min. The differential cell count was carried out using a digital light microscope (Olympus, USA) at 100x magnification by oil immersion technique. At least 200 cells were differentiated on each slide.

### Histopathology Using Hematoxylin and Eosin (H&E) and Sirius Red and Fast Green (SRFG) Stains

Standard techniques were employed for histopathological studies using H&E for morphological analysis (Aravamudan et al., 2012; Raju et al., 2014; Liu et al., 2017; Ambhore et al., 2019a; Britt et al., 2019). For SRFG staining to detect collagen deposition standard technique was followed as per manufacturer’s instructions (Chrondex, Inc., USA). Stained sections were scanned using Motic Easy Scan (Motic, Canada). Regions of interest were captured on the acquired H&E stained images (20X) using Motic DS Assistant Lite software (Motic, Canada) followed by analyzing them for ASM thickness using image J macros (Image j, NIH, USA). ROI’s in SRFG stained sections were subjected to a blind review and scored on a scale of 1 to 5 with one being minimal collagen deposition and five being maximum collagen deposition by three independent persons.

### Immunofluorescence

Paraffin embedded tissue blocks of mice lungs were sectioned into 6 µm thick sections using a microtome and transferred onto a slide. Slides were then incubated at 56°C for 2 h (De-paraffinization). Following this, antigen retrieval was performed by steaming the slides for 40 min using Sodium Citrate Buffer and left to cool down to room temperature for 30 min. Sections were washed with PBS, permeabilized using 0.1% TritonX-100 in PBS for 15 min and blocked using 10% goat serum for 1 hour at room temperature. Following blocking, sections were incubated with fibronectin (sc-9068, Santa Cruz Biotech, USA), vimentin (V5255, Sigma Aldrich, USA) and α-smooth muscle actin (A2547, Sigma Aldrich, USA) antibodies overnight at 4°C. The following day, sections were washed with PBS and incubated with either AF-488 or AF-555 tagged secondary antibodies raised against mouse or rabbit for 1 hour at room temperature. The slides were then washed with PBS and mounted with coverslips using mounting media loaded with DAPI. Control slides were subjected to the same process, except for the addition of primary antibody. Images were captured using Lionheart Fx imaging station (Biotek, USA) at 10X magnification. The obtained images were analyzed for total fluorescence intensity for each protein (Fibronectin, α-smooth muscle actin and Vimentin) using ImageJ 1.50i version (NIH).

### Statistical Analysis

A total of 72 mice were used for this study. Wild type, ERα KO and ERβ KO mice of C57BL/6J background (24 mice in each group) were sub-divided into two separate subpopulations based on gender (12 males and 12 females). Each sub-population was further divided into two groups: PBS and MA challenged group consisting of six mice in each group. Overall, the study was performed focused on 3 variables: 1) genetic background (WT, ERα KO and ERβ KO); 2) gender (Male and Female) and 3) disease condition (PBS and MA-induced asthma). “n” values represent number of animals. Statistical analysis was performed using two-way ANOVA followed by Bonferroni post-hoc multiple comparisons using GraphPad Prism version 8.1.0 for Windows (GraphPad Software, San Diego, California USA, www.graphpad.com). All data are expressed as mean ± SEM. Statistical significance was tested at the minimum of p < 0.05 level.

## Results

### Role of Differential ER Signaling on Airway Resistance (Rrs)

Role of differential ER signaling in mouse lung *in vivo* was determined using the flexiVent FX1 module with an in-line nebulizer (SciReq, Montreal, Canada). The experimental plan and confirmation of genotyping (representative images) are shown in **Figures 1A**, **B** and C respectively. Mice from all three-study populations (WT, ERα, and ERβ KO) showed a dose-dependent effect in lung function parameters after MCh challenge.

**Figure 2:**
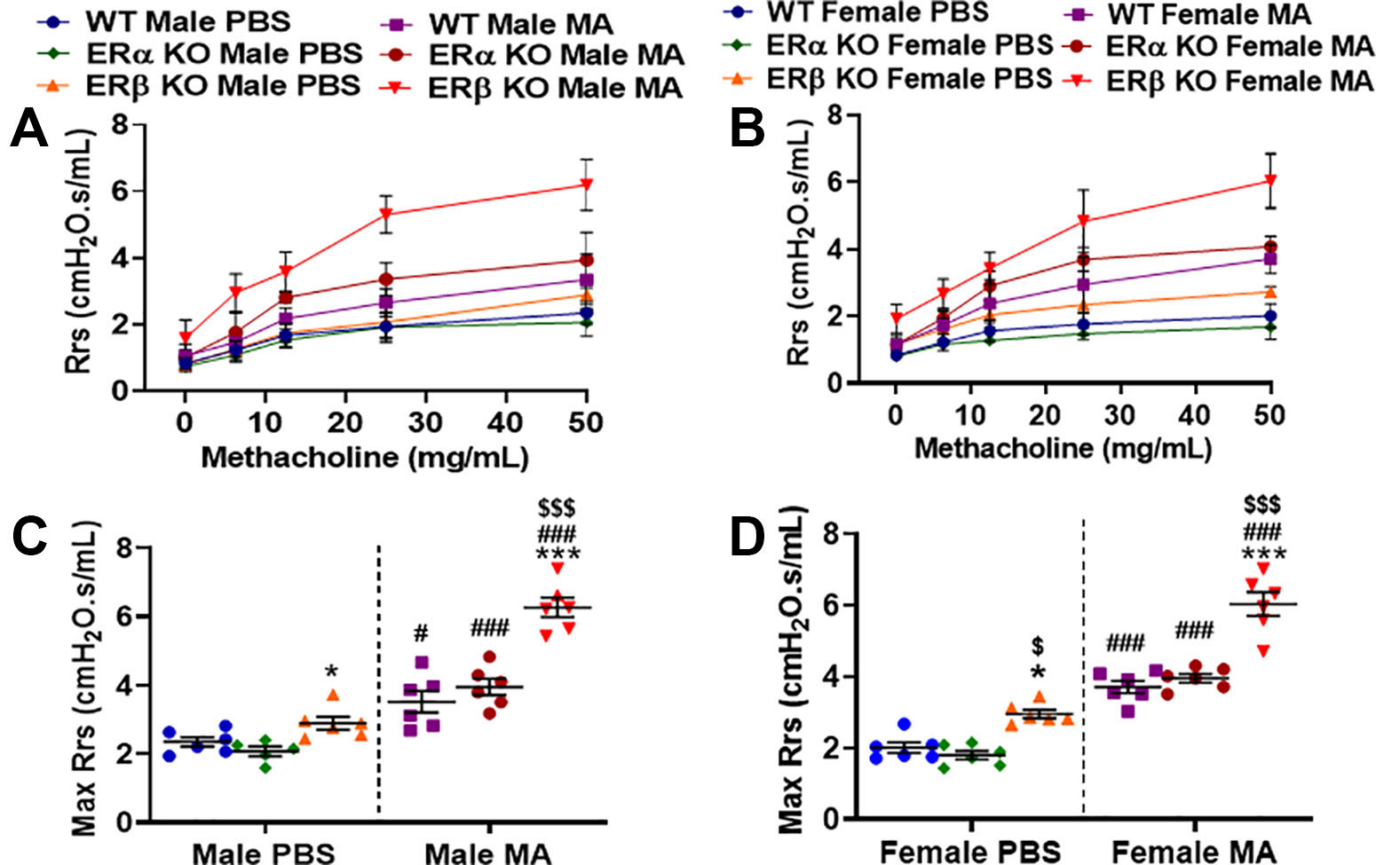
Effect of estrogen receptor (ER) signaling on airway resistance (Rrs) in the lungs of **(A)** male and **(B)** female mice (wild type, WT vs. ERα KO vs. ERβ KO) exposed to phosphate buffered saline (PBS) and mixed allergen (MA). Max Rrs was used to compare WT, ERα KO and ERβ KO in **(C)** males and **(D)** female mice exposed to PBS and MA. Data represented as mean ± SEM of at least 5-6 mice per treatment group; ^#^p < 0.05, ^###^p < 0.001 vs. PBS of respective groups (MA effect), *p < 0.05, ***p < 0.001 vs. WT of PBS/MA (ER specific KO effect) and ^$^p < 0.05, ^$$$^p < 0.001 vs. ERα KO of PBS/MA (ERβ vs. ERα effect).

Rrs indicates the dynamic resistance of the airways and quantitatively assesses the level of constriction in the lungs. Male and female mice from all three-study populations (WT, ERα KO and ERβ KO) showed a significant increase in Rrs after MCh challenge in MA exposed groups compared to PBS (**Figures 2A–D**). ERβ KO male and female (p < 0.05) mice showed a significant increase in Rrs compared to WT mice at baseline, while ERα KO mice (male and female) did not show any changes in the Rrs at baseline, rather showed a slight but not significant decrease compared to WT mice. MA challenged mice from all three study populations showed a significant increase in Rrs in males (p < 0.05 for WT; p < 0.001 for ERα KO and ERβ KO) and in females (p < 0.001 for WT, ERα KO and ERβ KO) compared to PBS challenged mice of respective populations with maximum changes observed in ERβ KO mice (both males and females; **Figures 2C**, **D**). Interestingly, ERβ KO females (p < 0.001) mice showed a significant increase in Rrs compared to ERα KO mice in the presence of MA (p < 0.001; **Figures 2C**, **D**).

### Role of Differential ER Signaling on Airway Compliance (Crs)

**Figure 3:**
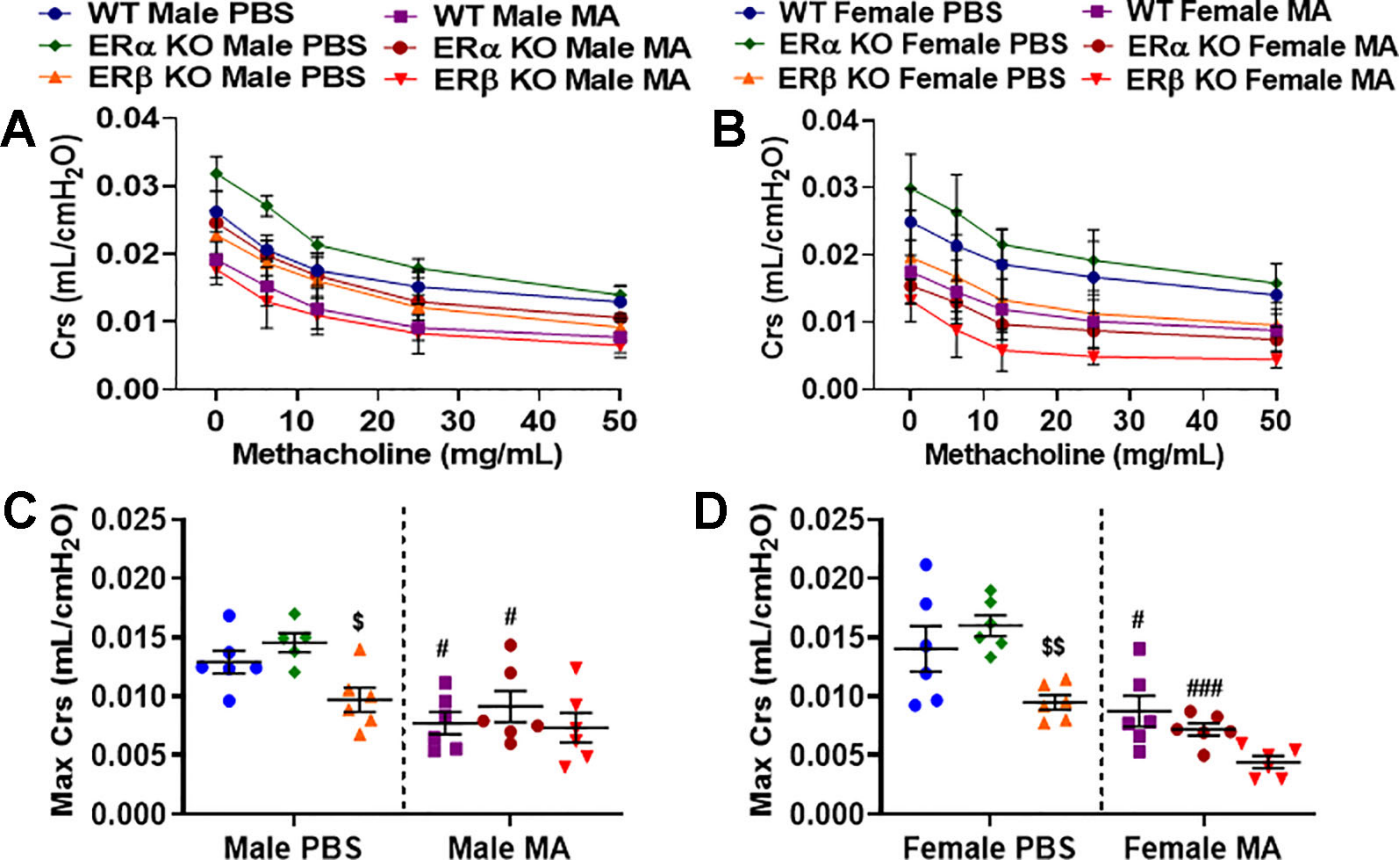
Effect of ER signaling on compliance (Crs) in the lungs of **(A)** male and **(B)** female mice (WT vs. ERα KO vs. ERβ KO) exposed to PBS and MA. Max Crs was used to compare WT, ERα KO and ERβ KO in **(C)** male and **(D)** female mice exposed to PBS and MA. Data represented as mean ± SEM of at least 5-6 mice per treatment group; ^#^p < 0.05, ^###^p < 0.001 vs. PBS of respective groups (MA effect) and ^$^p < 0.05, ^$$^p < 0.01 vs. ERα KO of PBS/MA (ERβ vs. ERα effect).

Crs depicts the ease with which respiratory system can be extended and provides insights into the overall elastic property of the respiratory system that is needed to overcome during tidal breathing and is inversely proportional to resistance. Male (p < 0.05) and female (p < 0.01) ERβ KO mice showed a significant decrease in compliance at baseline compared to ERα KO, whereas no significant changes were observed when compared to WT mice. ERα KO mice did not show any significant changes compared to WT mice (**Figures 3A**–**D**). All three study populations (WT, ERα KO and ERβ KO) challenged with MA showed significant decrease in Crs in males (p < 0.01 for WT and ERα KO) and in females (p < 0.05 for WT and p < 0.001 for ERα KO) compared to respective PBS challenged mice (**Figures 3C, D**). Notably, due to the decreased Crs in ERβ KO mice at baseline, we did not see any significant changes in Crs following MA challenge in either gender.

### Role of Differential ER Signaling on Airway Elastance (Ers)

**Figure 4:**
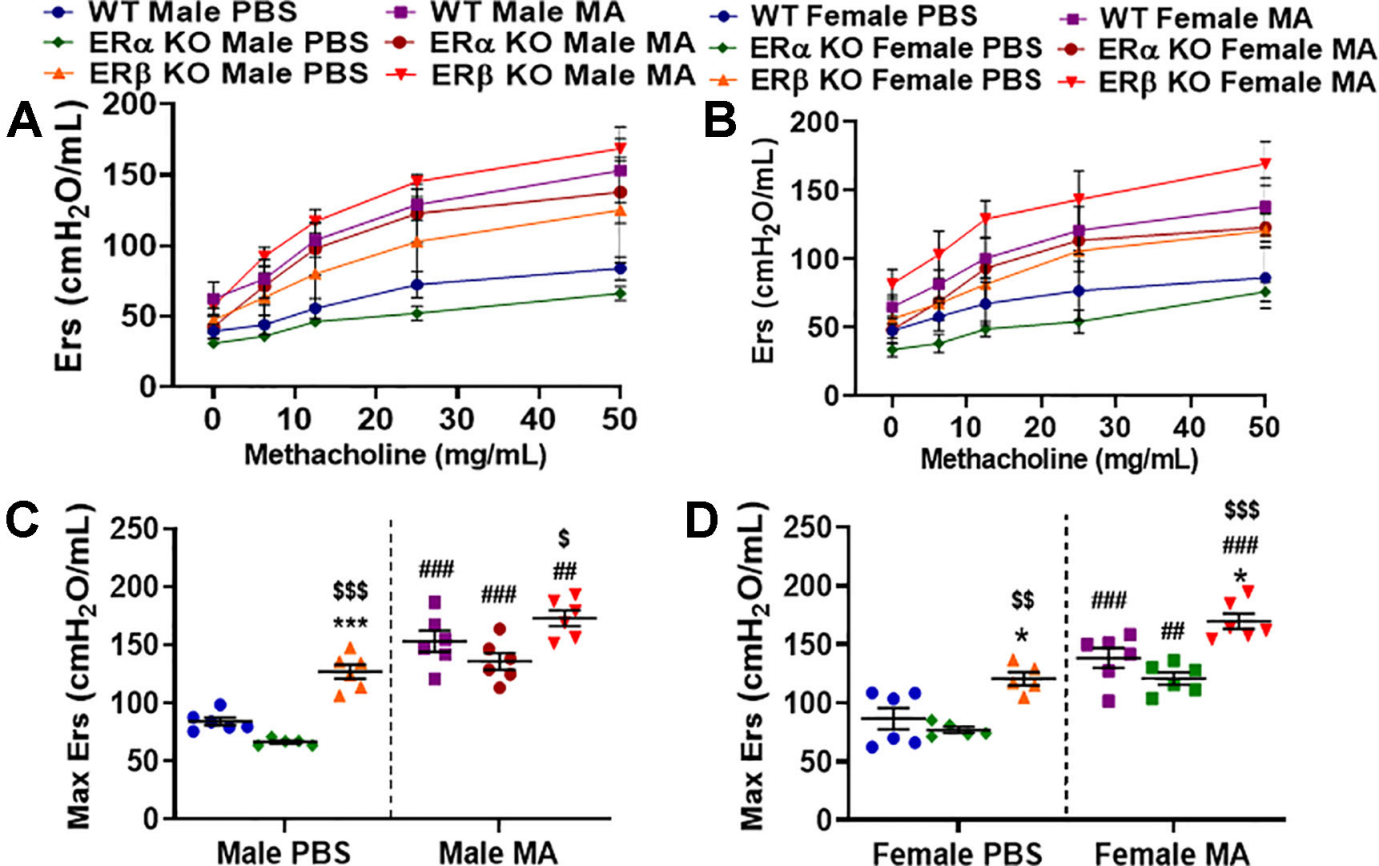
Effect of ER signaling on elastance (Ers) in the lungs of **(A)** male and **(B)** female mice (WT vs. ERα KO vs. ERβ KO) exposed to PBS and MA. Max Ers was used to compare WT, ERα KO and ERβ KO in **(C)** male and **(D)** female mice exposed to PBS and MA. Data represented as mean ± SEM of at least 5-6 mice per treatment group; ^##^p < 0.01, ^###^p < 0.001 vs. PBS of respective groups (MA effect), *p < 0.05, ***p < 0.001 vs. WT of PBS/MA (ER specific KO effect) and ^$^p < 0.05, ^$$^p < 0.01, ^$$$^p < 0.001 vs. ERα KO of PBS/MA (ERβ vs. ERα effect).

Airway elastance is related to the elastic stiffness of the respiratory system following an inhaled dose of MCh. Ers is often considered the reciprocal of Crs and vice versa and it captures the elastic stiffness of the airway. Ers also depicts the energy conservation in the alveoli to relax to a normal state. Ers was significantly increased at baseline in ERβ KO male (p < 0.001) and female mice (p < 0.05) compared to respective WT mice (**Figures 4A**–**D**). Furthermore, a significant increase in Ers was observed in ERβ KO male (p < 0.001) and female mice (p < 0.01) at baseline compared to ERα KO mice. Moreover, MA challenge has significantly increased Ers in males (p < 0.01 for ERβ KO and p < 0.001 for WT and ERα KO) and females (p < 0.01 for ERα KO, p < 0.001 for ERβ KO and WT) compared to PBS challenged mice across all three study populations with a maximum increase observed in WT males and females and ERα KO males (**Figures 4C**, **D**). ERβ KO mice challenged with MA showed significant increase in Ers in males (p < 0.05) and females (p < 0.001) compared to MA challenged ERα KO mice.

### Role of Differential ER Signaling on Tissue Damping (G)

**Figure 5:**
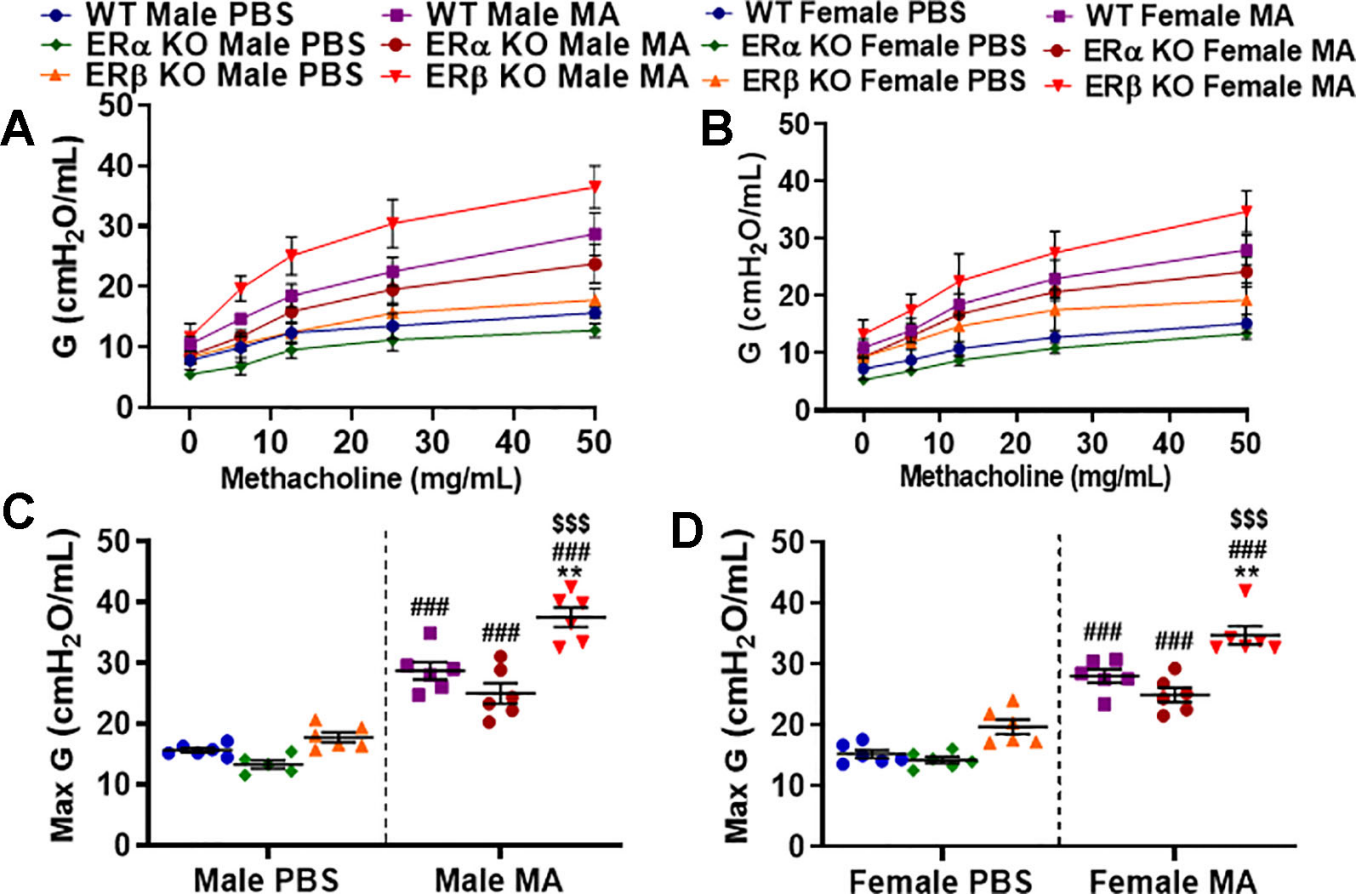
Effect of ER signaling on tissue damping (G) in the lungs of **(A)** male and **(B)** female mice (WT vs. ERα KO vs. ERβ KO) exposed to PBS and MA. Max G was used to compare WT, ERα KO and ERβ KO in **(C)** male and **(D)** female mice exposed to PBS and MA. Data represented as mean ± SEM of at least 5-6 mice per treatment group; ^###^p < 0.001 vs. PBS of respective groups (MA effect), **p < 0.01 vs. WT of PBS/MA (ER specific KO effect) and ^$$$^p < 0.001 vs. ERα KO of PBS/MA (ERβ vs. ERα effect).

Tissue damping (G) is a parameter of the constant phase model (CPM) that depicts energy dissipation in the alveoli. It is closely related to tissue resistance and will increase with the contraction of the ASM. MA challenged mice from all three study populations showed a significant increase in G in males and females (p < 0.001 for WT, ERα KO, and ERβ KO) compared to PBS challenged mice with maximum changes observed in ERβ KO mice (both males and females; **Figures 5A**–**D**). Moreover, ERβ KO male and female mice showed a significant increase (p < 0.001) in G compared to ERα KO and (p < 0.01) compared to WT mice in the presence of MA. Interesting finding here is neither ERβ KO nor ERα KO mice showed any significant changes in G at baseline condition compared to WT mice (**Figures 5C**, **D**). No changes in G were observed at baseline.

### Role of Differential ER Signaling on Tissue Elastance (H)

**Figure 6:**
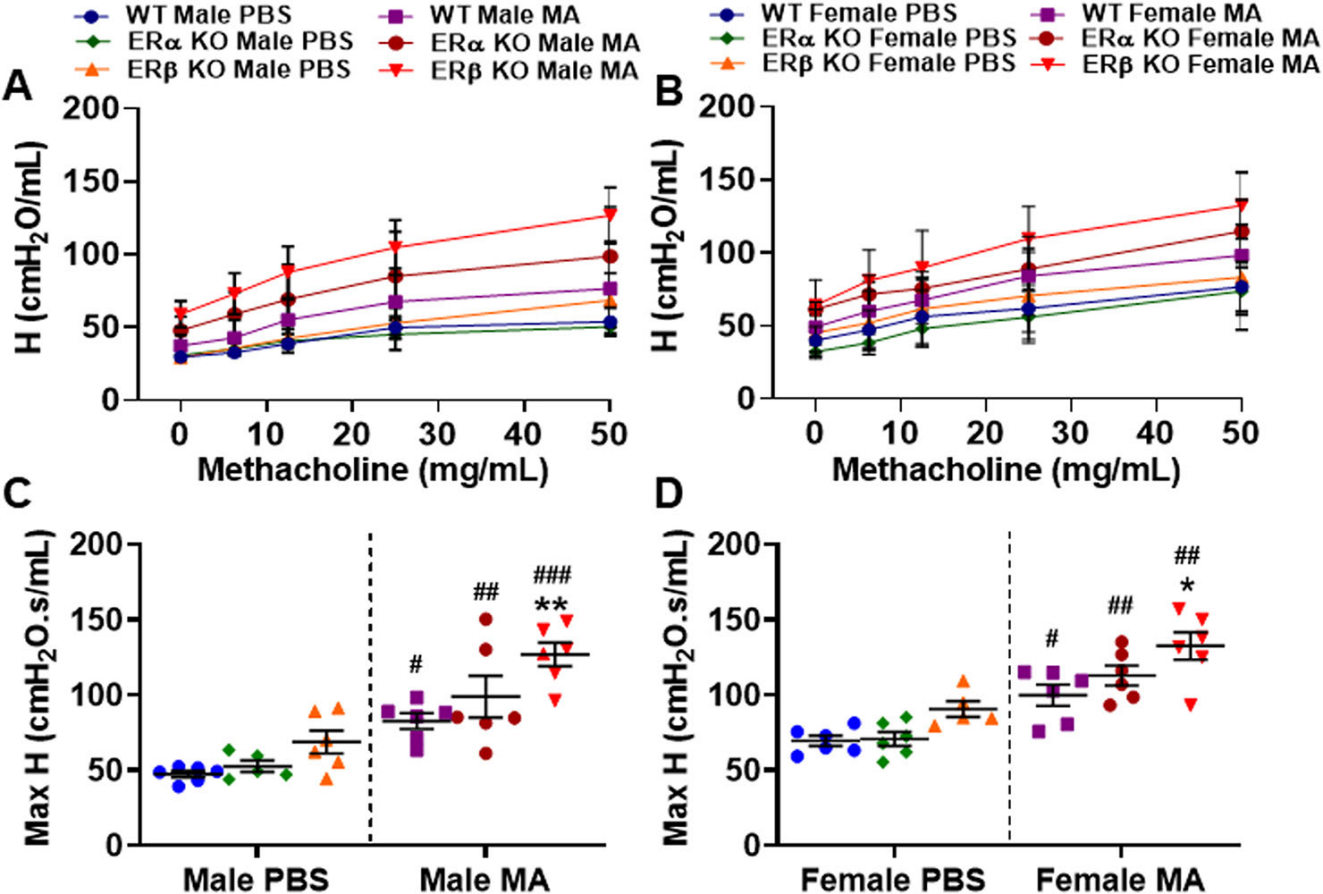
Effect of ER signaling on tissue elasticity (H) in the lungs of **(A)** male and **(B)** female mice (WT vs. ERα KO vs. ERβ KO) exposed to PBS and MA. Max H was used to compare WT, ERα KO and ERβ KO in **(C)** male and **(D)** female mice exposed to PBS and MA. Data represented as mean ± SEM of at least 5-6 mice per treatment group; ^#^p < 0.05, ^##^p < 0.01, ^###^p < 0.001 vs. PBS of respective groups (MA effect) and *p < 0.05, **p < 0.01 vs. WT of PBS/MA (ER specific KO effect).

The extent of energy conservation in the alveoli is depicted by tissue elastance (H) and it also indicates the elastic recoil of the lung or tissue stiffness that permits its return towards an initial form. Tissue elastance was significantly increased in male (p < 0.05 for WT, p < 0.01 for ERα KO and p < 0.001 for ERβ KO) and female (p < 0.05 for WT, p < 0.01 for ERα KO and ERβ KO) mice challenged with MA compared to respective PBS challenged mice across all three study populations (**Figures 6A**–**D**). The extent of increase in H upon MA challenge was found to be highest in ERβ KO male mice compared to all other groups. Furthermore, ERβ KO mice challenged with MA showed significant increase in H in males (p < 0.01) and females (p < 0.05) compared to MA challenged WT mice. Similar to G, neither ERβ KO nor ERα KO mice showed any significant changes in the H at baseline condition compared to WT mice (**Figures 6C**, **D**). Moreover, ERβ KO mice of either gender did not show any significant changes in H when compared to ERα KO mice.

### Effect of ER Signaling on Total and Differential Leukocyte Count

**Figure 7:**
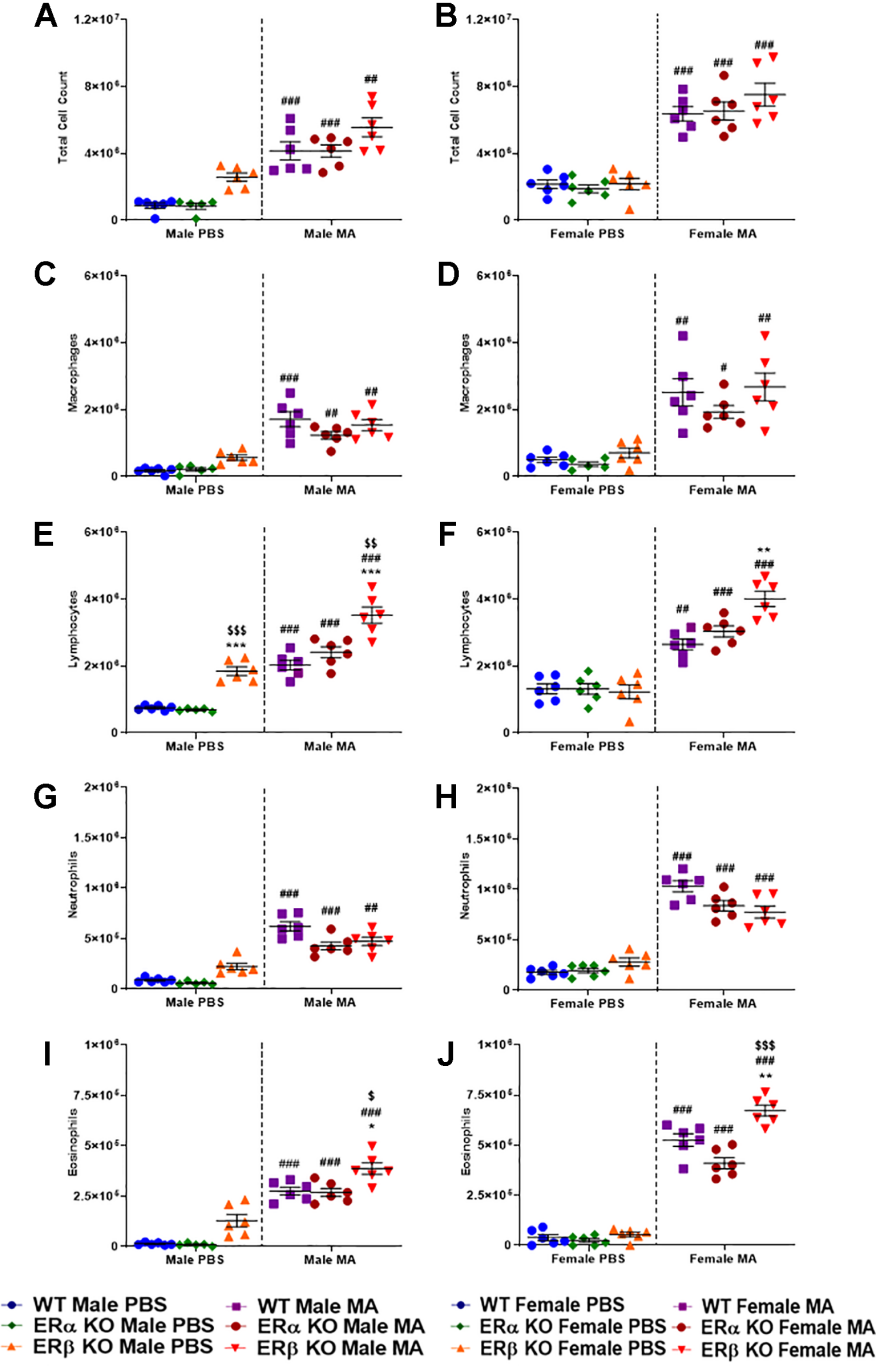
Effect of ER signaling on total and differential leukocyte count. Dot blots showing total cell count in **(A)** males and **(B)** females; Macrophage count in **(C)** males **(D)** females; Lymphocyte count in **(E)** males and **(F)** females; Neutrophils count in **(G)** males and **(H)** females and Eosinophils count in **(I)** males and **(J)** females. Data represented as mean ± SEM of at least 6 mice per treatment group; ^#^p < 0.05, ^##^p < 0.01, ^###^p < 0.001 vs. PBS of respective groups (MA effect) and *p < 0.05, **p < 0.01 vs. WT of PBS/MA (ER specific KO effect) and ^$^p < 0.05, ^$$^p < 0.01, ^$$$^p < 0.001 vs. ERα KO of PBS/MA (ERβ vs. ERα effect).

At baseline (PBS), WT and ERα KO mice of either gender did not show any significant changes in total or differential cell count; however, a notable increase in total and differential count was observed in ERβ KO mice, especially males, when compared to WT mice, although not significant. Mice from all three study populations showed a significant increase (p < 0.001 for WT and ERα KO, and p < 0.01 for ERβ KO in males; p < 0.001 for WT, ERα KO and ERβ KO in females) in the total cell count upon MA challenge when compared to respective PBS challenged mice (**Figures 7A**, **B**). Macrophage count in the BALF was significantly increased upon MA challenge (p < 0.001 for WT males, p < 0.01 for ERα KO and ERβ KO males; p < 0.01 for WT and ERβ KO females, p < 0.05 for ERα KO females) compared to respective PBS challenged mice (**Figures 7C**, **D**). At baseline, ERβ KO males showed significant increase in lymphocyte count compared to WT males (p < 0.001) and ERα KO males (p < 0.001). Lymphocyte count was significantly increased upon MA challenge (p < 0.001 for WT, ERβ KO and ERα KO males; p < 0.01 for WT females and p < 0.001 for ERα KO and ERβ KO females) compared to respective PBS challenged mice (**Figures 7E**, **F**). Furthermore, a significant increase in lymphocyte count was observed in MA challenged ERβ KO mice compared to MA challenged WT mice (p < 0.001) and MA challenged ERα KO mice (p < 0.01). Recruitment of neutrophils was significantly increased upon MA challenged mice (p < 0.001 for WT and ERα KO males, p < 0.01 for ERβ KO males; p < 0.001 WT, ERα KO and ERβ KO females) compared to respective PBS challenged mice (**Figures 7G**, **H**). Eosinophilic infiltration was significantly increased in MA challenged mice (p < 0.001 for WT, ERα KO and ERβ KO males and females) compared to respective PBS challenged mice (**Figures 7I**, **J**). Notably, MA challenged ERβ KO mice showed a significant increase in eosinophil count compared to MA challenged WT mice (p < 0.05 for males and p < 0.01 for females) as well as MA challenged ERα KO mice (p < 0.05 for males and p < 0.001 for females).

### Effect of ER Signaling on Lung Histology

**Figure 8:**
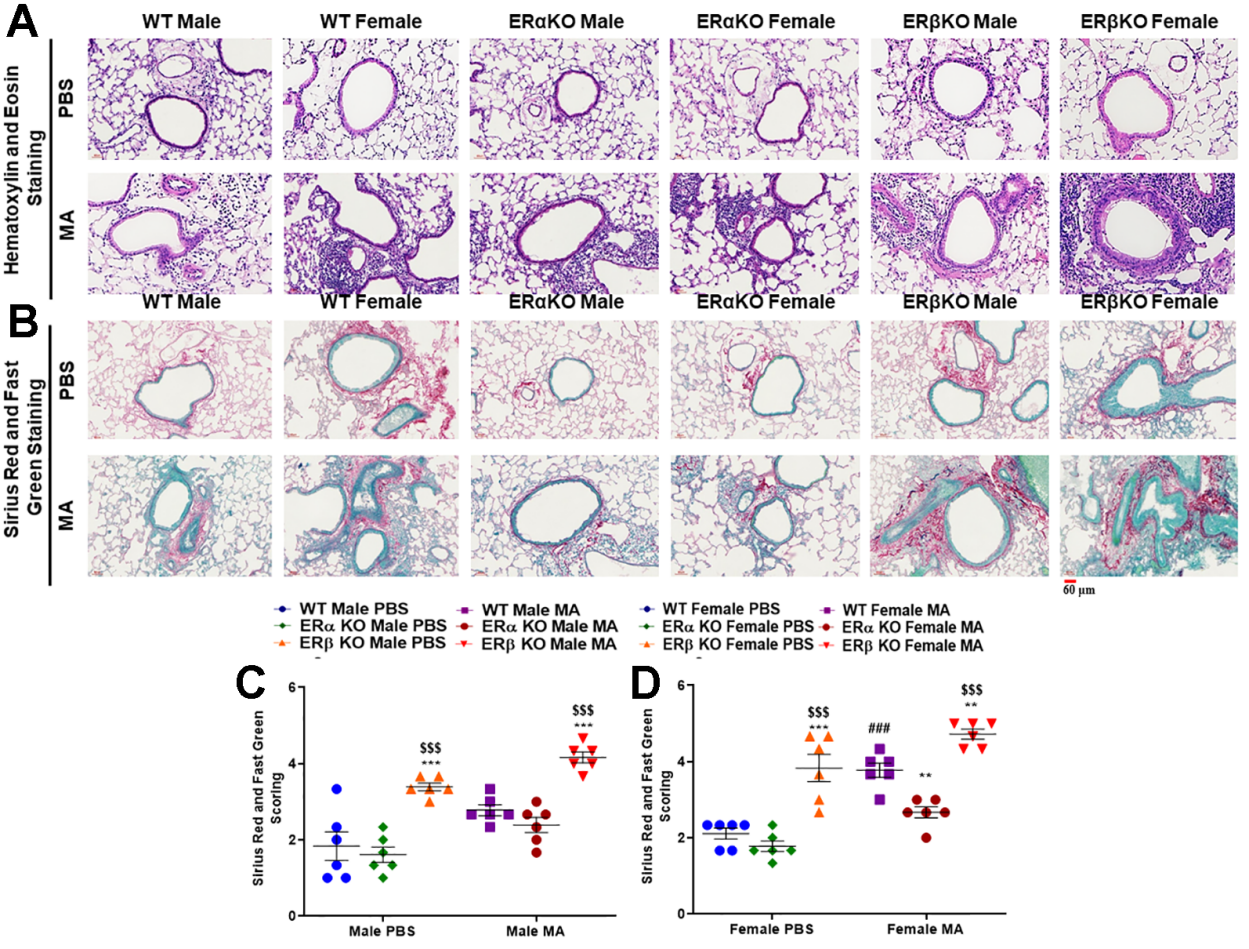
**(A)** H&E stained mice lung sections show increased thickness of the airway epithelium and ASM layer in MA challenged WT and ERβ KO male and female mice, while ERα KO mice did not show any prominent changes. In addition, the infiltration of inflammatory cells was increased in the airways of MA challenged WT, ERα KO and ERβ KO mice (both male and female), with a robust increase observed in ERβ KO mice. **(B)** Sirius red and fast green stained lung sections showing increased collagen deposition (green staining) in the airways of MA challenged WT, ERα KO and ERβ KO mice (both male and female). Scoring values of SRFG stained sections of **(C)** males and **(D)** females showing the extent of collagen deposition. Data represented as mean ± SEM of at least 6 mice per treatment group; ^###^p < 0.001 vs. PBS of respective groups (MA effect), **p < 0.01, ***p < 0.001 vs. WT of PBS/MA (ER specific KO effect) and ^$$$^p < 0.001 vs. ERα KO of PBS/MA (ERβ vs. ERα effect).

H&E staining showed increased thickness of the airway epithelium and ASM layer in MA challenged WT, ERα KO and ERβ KO male and female mice compared to respective PBS challenged mice, with higher magnitude observed in ERβ KO female mice (**Figure 8A**). Furthermore, the infiltration of inflammatory cells was increased in the airways of MA challenged WT, ERα KO and ERβ KO mice (both males and females), with a robust increase in inflammatory cells was observed in ERβ KO mice (**Figure 8A**). In addition, Sirius red and fast green (SRFG) stained lung sections showed significant increase in collagen deposition (red staining) in the airways of ERβ KO male (p < 0.001 vs. WT and ERα KO) and female (p < 0.01 vs. WT and p < 0.001 vs. ERα KO) mice at baseline (PBS) (**Figures 8C**, **D**). MA challenged WT mice showed a significant increase in collagen deposition (p < 0.001) in females but not in males compared to PBS challenged mice. ERα KO and ERβ KO female mice (both male and female) compared to respective PBS challenged mice with profound changes observed in ERβ KO mice (**Figures 8C**, **D**).

### Effect of ER Signaling on Fibronectin, Vimentin, and α-SMA

**Figure 9:**
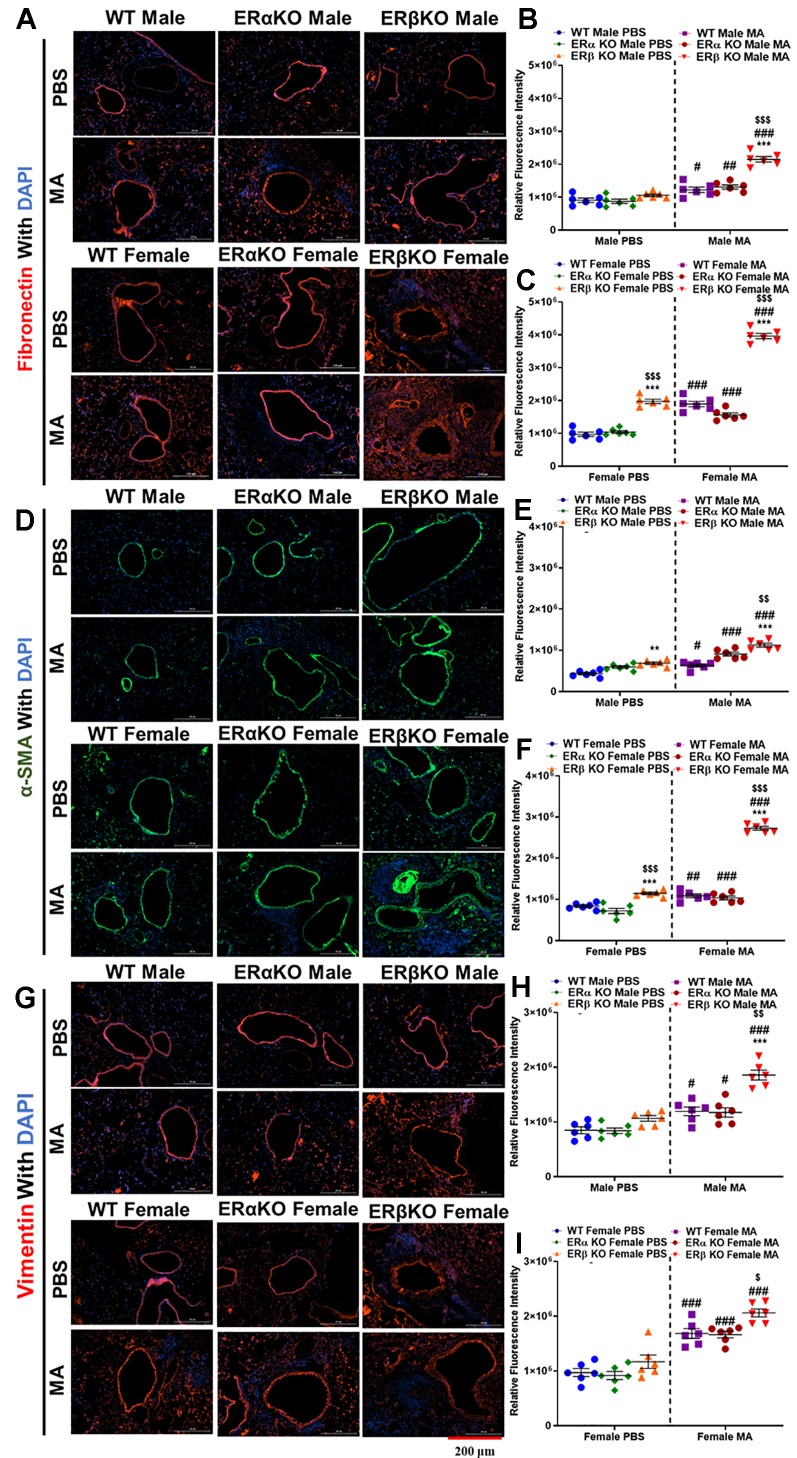
Effect of ER signaling on expression of fibronectin, α-SMA and vimentin. Representative images of airway sections probed with fibronectin **(A)**, quantification of relative fluorescence intensity (RFI) of fibronectin in males **(B)** and females **(C)**. Representative images of airway sections probed with α-smooth muscle actin (α-SMA, **D**), quantification of RFI of α-SMA in males **(E)** and females **(F)**. Representative images of airway sections probed with vimentin **(G)**, quantification of RFI of vimentin in males **(H)** and females **(I)**. Data in b, c, e, f, h and i represented as mean ± SEM of at least 6 mice per treatment group. ^#^p < 0.05, ^##^p < 0.01, ^###^p < 0.001 vs. PBS of respective groups (MA effect) and **p < 0.01, ***p < 0.001 vs. WT of PBS/MA (ER specific KO effect) ^$^p < 0.05, ^$$^p < 0.01, ^$$$^p < 0.001, vs. ERa KO of PBS/MA (ERb vs. ERa effect).

Fibronectin, vimentin, and α-SMA are characteristic markers for ECM deposition, fibrotic changes in the lungs and ASM phenotype respectively. At baseline, WT and ERα KO mice of either gender did not show any significant changes in fibronectin, vimentin and α-SMA. However, ERβ KO mice showed significantly increased fibronectin (p < 0.001 for females) and α-SMA (p < 0.005 for males and p < 0.001 for females), but not vimentin at baseline compared to WT mice.Moreover, ERβ KO female mice showed a significant increase in fibronectin (p < 0.001) and αSMA (p < 0.001) compared to ERα KO mice at baseline. MA challenge significantly increased fibronectin (p < 0.05 for WT, p < 0.005 for ERα KO and p < 0.001 for ERβ KO in males; p < 0.001 for WT, ERα KO and ERβ KO in females), α-SMA (p < 0.05 for WT, p < 0.001 for ERα KO and ERβ KO in males; p < 0.005 for WT, p < 0.001 for ERα KO and ERβ KO in females) and vimentin (p < 0.05 for WT and ERα KO, p < 0.001 for ERβ KO in males; p < 0.001 for WT, ERα KO and ERβ KO in females) compared to respective controls (**Figures 9A**–**I**).

## Discussion

Airway inflammation, remodeling and AHR are considered as cardinal features of asthma leading to obstruction of airways (Holgate et al., 2009; Prakash, 2013). Asthma is a multifaceted and intricate disease involving diverse pathologies, which makes it challenging to identify and address the core mechanisms involved (Hershenson et al., 2008; Raju et al., 2014; Sathish et al., 2015b; Prakash, 2016). Akin to clinical data, sex differences and sex steroids play a crucial role in the incidence and severity of asthma (Clough, 1993; Redline and Gold, 1994; Weiss and Gold, 1995; Caracta, 2003; Carey et al., 2007c; Card and Zeldin, 2009; Antunes et al., 2010; Woods et al., 2010; Townsend et al., 2012a; Bonds and Midoro-Horiuti, 2013; Sathish et al., 2015b; Sathish and Prakash, 2016). Considering the fact that women are more prone to the occurrence of asthma than men (de Marco et al., 2000; Melgert et al., 2005; Carey et al., 2007b; Matsubara et al., 2008; Vink et al., 2010; Dursun et al., 2014; Fuseini and Newcomb, 2017; Pignataro et al., 2017; Han et al., 2018), identifying the role of sex steroids, especially estrogen in airways might shed some light on the pathology of asthma.

Estrogen has a systemic role beyond the reproductive system and the evidence suggests a wide array of roles for estrogen in both males and females in regulating cell growth and differentiation, intracellular calcium regulation and inflammation (Townsend et al., 2010; Townsend et al., 2012a; Townsend et al., 2012b; Sathish et al., 2015a; Sathish and Prakash, 2016). Given the facts about estrogen and the lack of consensus whether it is pro-inflammatory vs. anti-inflammatory and to define the consequences in structural cells of the airways, it is important to understand the mechanisms involved in estrogen signaling. In order to identify the role of estrogen signaling in asthma *in vivo*, multiple studies have been performed in the past; however, none of them have been able to provide a complete picture (Carey et al., 2007a; Carey et al., 2007c; Riffo-Vasquez et al., 2007; Jia et al., 2011; Itoga et al., 2015; El-Desouki et al., 2016). Most of these studies have largely focused on the role of estrogen *per se* (Carey et al., 2007c; El-Desouki et al., 2016), but did not focus on the receptor specific effects involved. Moreover, very limited data is available on the receptor-based mechanisms of estrogen *in vivo*, which are either based on Penh or focused on ERα, completely disregarding ERβ (Carey et al., 2007a; Riffo-Vasquez et al., 2007).

In our previous studies, we showed differential expression of ER’s in asthmatics and nonasthmatics, which is upregulated (especially ERβ) during asthma or inflammation (Aravamudan et al., 2017). Furthermore, we also showed that activation of ERβ (using an ERβ specific pharmacological agonist, WAY200070) downregulated human ASM proliferation *in vitro* (Ambhore et al., 2018). In addition, our recent *in vivo* study also shows that ERβ activation using pharmacological agonist downregulated AHR and remodeling in MA challenged WT mice (Ambhore et al., 2019a). Although pharmacological agonists used in our earlier study had high affinity to their designated receptors, there is still a minor possibility of cross-reactivity. In connection to this, in order to establish the comprehensive role of physiological estrogen in the airways and to avoid the cross-reactivity of the pharmacological receptor agonists, we employed ERα KO and ERβ KO mice, which will provide valuable insights into the receptor-based effects of endogenous estrogen on AHR and airway remodeling.

In this study, we employed MA induced model of asthma, as it is considered to be robust and the most effective model of mimicking human asthma in murine models (Aravamudan et al., 2012; Iijima et al., 2014; Yarova et al., 2015; Ambhore et al., 2019a; Britt et al., 2019). Airway mechanics were determined using the forced oscillation technique (FOT) of the FlexiVent Fx1 module, which is an invasive endpoint technique that delivers parameters like airway resistance (Rrs), compliance (Crs), elastance (Ers), tissue elasticity (H) and tissue damping (G), which together depict the overall lung function (Aravamudan et al., 2012; Ambhore et al., 2019a). Our study shows that ERβ KO mice show deteriorated lung function compared to WT and ERα KO in both the genders at baseline, with prominent changes observed in females compared to males, which correlates with earlier clinical findings suggesting females are susceptible to asthma (Weiss and Gold, 1995; Angele et al., 2000; de Marco et al., 2000; Caracta, 2003; Melgert et al., 2005; Carey et al., 2007b; Matsubara et al., 2008; Takeda et al., 2013; Han et al., 2018). Interestingly, ERα KO mice of either sex showed no changes in lung function compared to WT mice at baseline, which can be attributed to the protective role of ERβ or detrimental role of ERα in the airways, especially in ASM (Ambhore et al., 2018; Ambhore et al., 2019a). In addition, MA challenged mice of all three populations (WT, ERα KO, and ERβ KO) showed a significant decline in lung function compared to respective PBS treated mice. Here, female mice exposed to MA in all three study populations showed prominent decline compared to males, which corroborates with clinical data suggesting increased severity of asthma in females (Weiss and Gold, 1995; de Marco et al., 2000; Melgert et al., 2005; Vink et al., 2010; Han et al., 2018). In addition, the severity of MA induced AHR and remodeling was found to be more pronounced in ERβ KO mice compared to WT and ERα KO. This corroborates with our previous findings where we have shown that activation of ERβ using WAY200070 (an ERβ selective agonist) resulted in improved lung function in WT mice challenged with MA compared to MA alone (Ambhore et al., 2019a).

One of the cardinal features of asthma is infiltration of inflammatory cells like lymphocytes, monocytes/macrophages, neutrophils and eosinophils into the airways, especially eosinophilic infiltration, which is associated with the development and aggravation of AHR (Gleich, 2000; Wardlaw et al., 1988; Gupta et al., 2014). Lymphocytes, both T and B play a crucial role in coordinating inflammatory response in asthma (Mosmann and Sad, 1996; Gould et al., 2000). T-lymphocytes are often considered to express a distinctive pattern of cytokines, especially Th2 cytokines, which contribute to remodeling and AHR (Mosmann and Sad, 1996); whereas, Blymphocytes secrete IgE and the factors regulating IgE secretion, which result in recruiting inflammatory cells into the airways, eventually contributing to airway inflammation (Gould et al., 2000). Neutrophils are not a predominant cell type observed in the airways of patients with mild to moderate chronic asthma, whereas they appear to be a more prominent cell type in the airways and induced sputum of patients with more severe asthma (Wenzel et al., 1997; Jatakanon et al., 1999; Gibson et al., 2001). Evidence suggests experimentally activated eosinophils induce airway epithelial damage (Yukawa et al., 1990). In this study, we found that MA exposure significantly increased the total and differential leukocyte count in the airways of mice, especially in females with prominent changes in ERβ KO mice which concurs with clinical evidence (Melgert et al., 2005; Blacquiere et al., 2010; Fuseini and Newcomb, 2017; Pignataro et al., 2017; DeBoer et al., 2018) and our own pre-clinical findings (Aravamudan et al., 2017; Ambhore et al., 2018; Ambhore et al., 2019a) indicating females are more susceptible to asthma and that ERβ plays a protective role in regulating inflammatory cell infiltration. Very little information is available on the ER specific effects on inflammatory cells in the lung during asthma, which warrants more in depth immune cell based studies in the future.

Histology studies using H&E stain show no prominent changes across all three-study populations at baseline; however, upon MA challenge significant changes in the thickness of the epithelium and ASM were observed in all three study populations (especially females), with maximum changes observed in ERβ KO mice, which corroborates with our earlier findings, where we showed ERβ activation downregulates ASM proliferation (Ambhore et al., 2018). Furthermore, the increase in inflammatory cells infiltration observed in ERβ KO mice challenged with MA concurs with our previous study, where ERβ activation resulted in reduced infiltration of inflammatory cells in the airways suggesting ERβ plays a crucial role in regulating inflammation (Ambhore et al., 2019a). In addition, SRFG stained lung sections showed increased collagen deposition in mice exposed to MA across all three-study populations with prominent changes observed in ERβ KO mice. Increased collagen deposition is an indicator for an increase in ECM deposition, which leads to airway remodeling (Hocking, 2002; Chakir et al., 2015). These observations are in accordance with previous clinical histology findings, which indicate increased epithelial dysplasia, blood vessels, ASM mass and extracellular matrix (ECM) deposition lead to airway wall thickening (Colvillenash et al., 1995; Burgess, 2009).

Fibronectin, like collagen is another ECM protein marker that indicates the extent of airway remodeling (Shoji et al., 1990; Shiels et al., 1999). Vimentin is considered as a marker for detecting fibrosis, which indicates phenotype changes in ASM to fibroblast (Wang et al., 2006; Wang et al., 2007; Slats et al., 2008; Tang and Gerlach, 2017); whereas, α-SMA is a smooth muscle specific marker, ASM in this case (Ambhore et al., 2019a; Britt et al., 2019; Loganathan et al., 2019). Immunofluorescence studies of mice lung sections indicate increased expression of fibronectin, vimentin and α-SMA in mice from all three-study populations exposed to MA, especially in ERβ KO mice, indicating significant remodeling of the airways in the absence of ERβ implicating a crucial role for ERβ in regulating airway remodeling.

In conclusion, the results from this study suggest the importance of ER’s and their signaling in the lungs and their role in regulating the overall lung function. In addition, this study implicates the differential role of ERα and ERβ in airway physiology during asthma, especially in the context of AHR and remodeling. Considering the “protective role of ERβ” during asthma, it is noteworthy to identify ERβ as a potential target to develop novel lead molecules that can be used as alternative therapies to treat asthma.

## Data Availability Statement

The datasets generated for this study are available on request to the corresponding author.

## Ethics Statement

The animal study was reviewed and approved by the Institutional Animal Care and Use Committee (IACUC) - North Dakota State University.

## Author Contributions 

RK, NA, and VS conceived the idea and laid out the plan of study. RK, NA, SB, and JL performed the experiments, calculations and statistical analysis. RK, NA, SB, JL, and VS prepared the manuscript.

## Funding

Supported by NIH grants R01 HL123494, R01 HL123494-02S1 (Venkatachalem). Additional support in part from ND EPSCoR with NSF #1355466.

## Conflict of Interest

The authors declare that the research was conducted in the absence of any commercial or financial relationships that could be construed as a potential conflict of interest.
